# Supplementary material for: Untwisting the Caenorhabditis elegans embryo
Source: eLife. 2015 Dec 3;4:e10070. doi: 10.7554/eLife.10070 (PMC4764590; doi:10.7554/eLife.10070)
Supplement: Supplementary file 1. — DOI: http://dx.doi.org/10.7554/eLife.10070.034 [file elife-10070-supp1.doc]

**Supplementary File 1, Tutorial for use of the WormUntwisting automated lattice-building plugin**.

**System Requirements:**

The plugin should work on most modern desktop PCs with a good graphics card. We have used it on the following systems with good results:

Operating system: Windows 7 professional

GPU card: Quadro K5000

Processor: Intel Xeon CPU E5-2630 v2 @ 2.60GHz (24 cores), Intel Core i7-2600 @3.40GHz

Memory (RAM): at least 6 GB

System Type: 64-bit (required)

**Downloading the Java Runtime Environment (64-bit required):**

1. Visit <http://www.java.com/en/download/manual.jsp>
2. Choose “Windows Offline (64-bit)” to download and install

**Downloading the plugin:**

1. The plugin is implemented as a JWS (Java Web Start) snapshot.

2. Navigate to <http://mipav.cit.nih.gov/plugin_jws/mipav_worm_plugin.php?mem_limit=8000> using a web browser. Select the option to save the file to disk, if no choice is presented the file is downloaded automatically.

3. Double-click on the mipav-worm-plugin JNLP file to open it.

4. Allow the program to access the computer. The program needs access to an active internet connection during the first run in order to download associated .jar files. After the first run the program can run without internet access.

5. The program will open to display the main menu.

**Plugin Overview:**

1. The WormUntwisting Plugin has three main components: a semi-automated segmentation, lattice-building, and untwisting component; a maximum-intensity-projection animation component; and an annotation animation component.

2. The semi-automated segmentation, lattice-building, and untwisting component is the base of the plugin. It allows a user to build lattices, straighten worm volumes, and annotate the image to determine 3D position after untwisting. The other two components use the output files generated by this component.

3. The maximum intensity projection animation component takes the straightened volumes and uses ImageJ to create a stack containing maximum intensity projections of all untwisted volumes. This feature requires ImageJ.

4. The annotation animation component uses 3D positional information contained in spreadsheet format to create a rendering showing the movement of each annotation point over time.

**Running the WormUntwisting plugin:**

1. The main menu will look as follows:


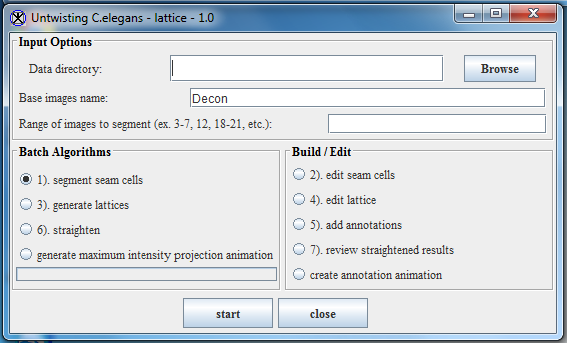


2. Select the directory containing the image sequence to be untwisted, and enter the base name of the image sequence. Set the number of images in the sequence using the “Range of images to segment” dialogue. If no numbers are entered the plugin will not work. An example:


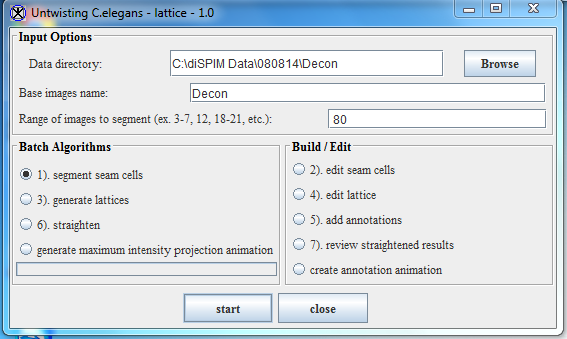


3. Select Option 1), segment seam cells, and press start to begin automatic segmentation of images in the sequence. The plugin will begin to automatically segment the images. Progress can be tracked via the progress bar on the lower left side of the plugin. When completed, a notification appears:


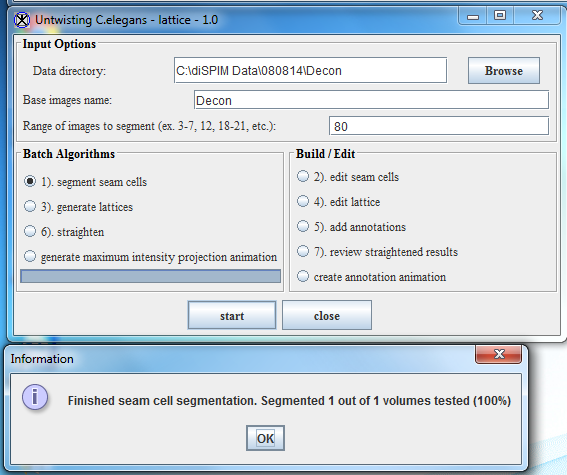


4. Select 2), edit seam cells, to check and edit the accuracy of the seam cell segmentations and to add a nose point. Doing so will open a window that looks like:


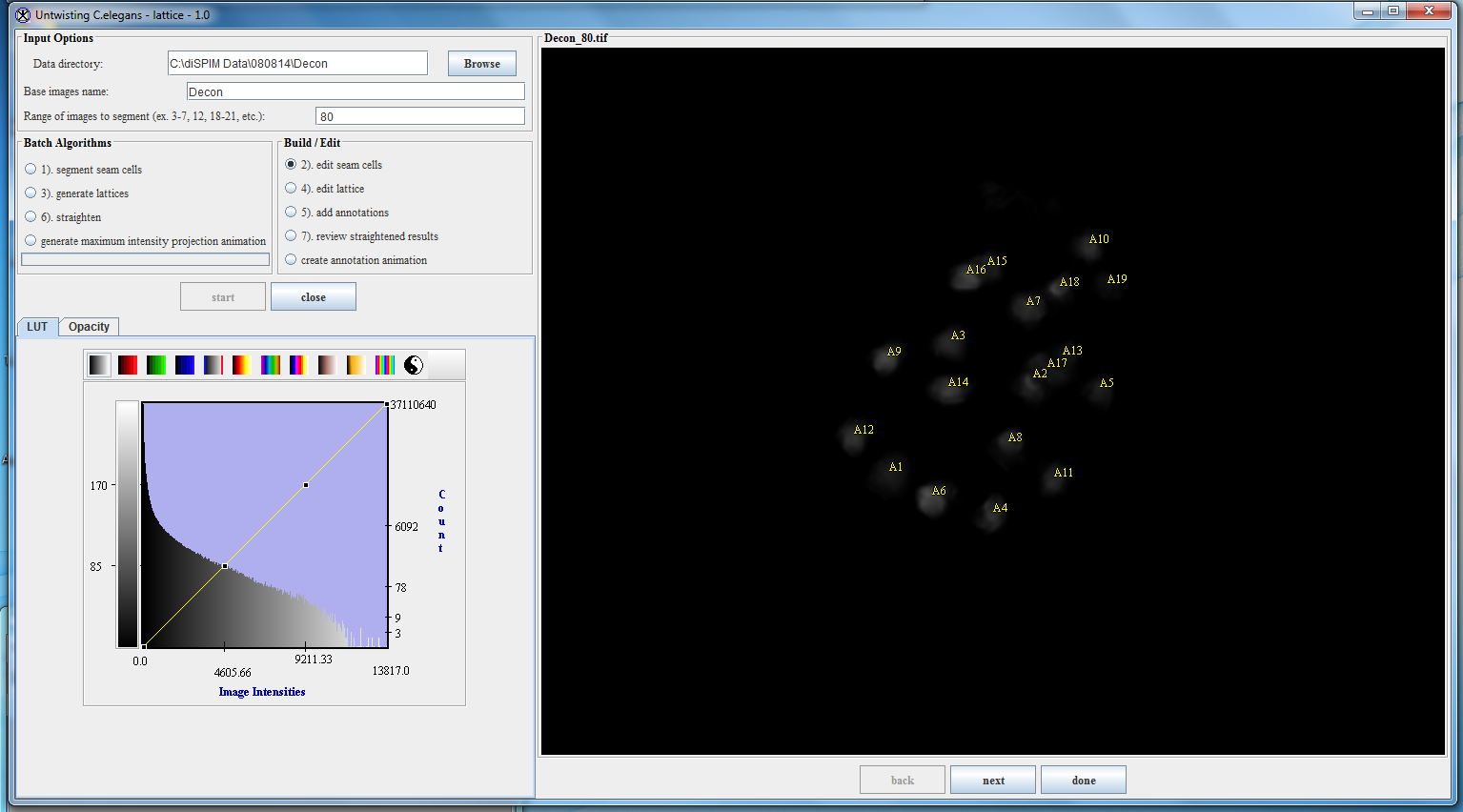


The window includes a large area for the Volume Renderer, which displays a 3D projection of the volume currently being worked on, the “back” and “next” buttons at the bottom allowing you to move through images in the sequence that is being edited, and a pair of LUT and Opacity histograms on the left side that are useful for adjusting the opacity of the rendered image volume.

5. Edit the segmentations for accuracy. Lattice-building assumes that 20 or 22 seam cell nuclei are present in the animal, but automated segmentation may over- or under-segment the image, resulting in an inaccurate number of seam cell nuclei. The number of seam cell nuclei segmentations is color-coded; less than 20 or 21 segmentations are shown with yellow labels, 20 or 22 are shown with green labels, and more than 22 are shown with red labels. To add segmentations control-click on a point within the Volume Renderer window and a new point will be placed where you click. To delete segmentations control-click on an existing segmentation then press the delete button on the keyboard.

6. Add or delete segmentations as necessary to reach a total of 20 or 22 seam cell nucleus segmentations, then add a final point at the nose of the animal. The edited segmentations should appear as:


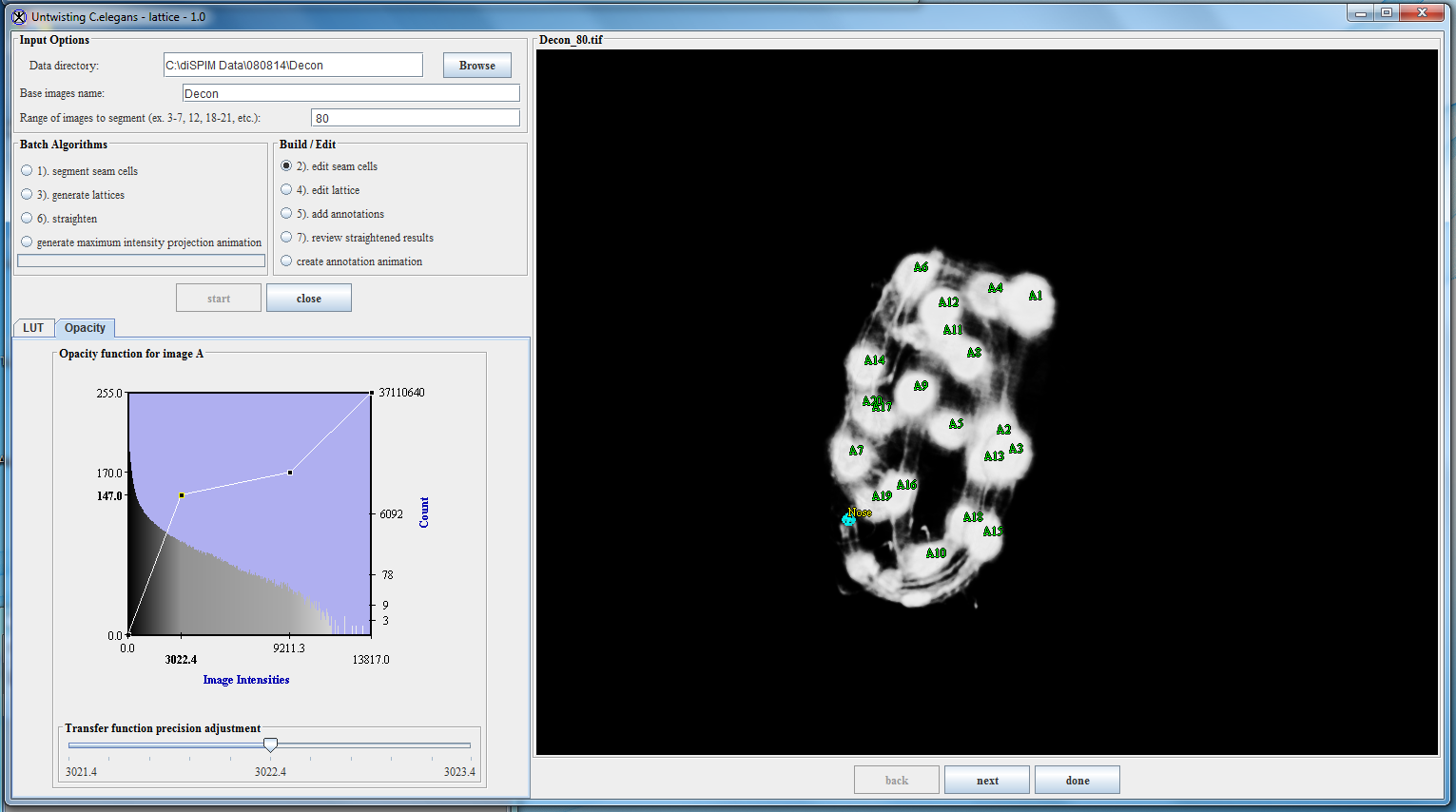


Press “done” when editing is completed.

7. Select 3), generate lattices, to start automated lattice generation:


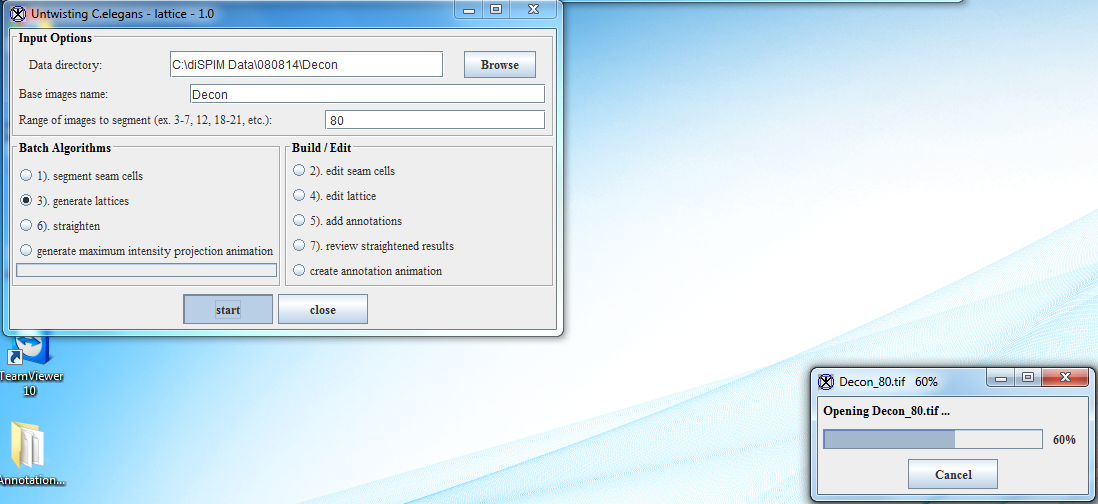


8. When lattice generation is finished, select 4), edit lattice. Automated lattice-generation automatically selects the top 5 lattices to display. You can select different lattices using the numbered buttons on the bottom. As with editing seam cell nuclei segmentations, the “back” and “next” buttons move through the images in the sequence. Once a lattice that matches the morphology of the animal has been identified, it can be further edited by moving pre-existing lattice points or adding new ones. To move a current lattice point, control-click on the point to select it and then either click and drag to move, or move by using the arrow keys. To add a new point, control-click on the desired location. Once added, points can be moved like pre-existing points. You can add and move points until the selected lattice matches the morphology of the embryo in the volume being worked on. An edited lattice appears like this:


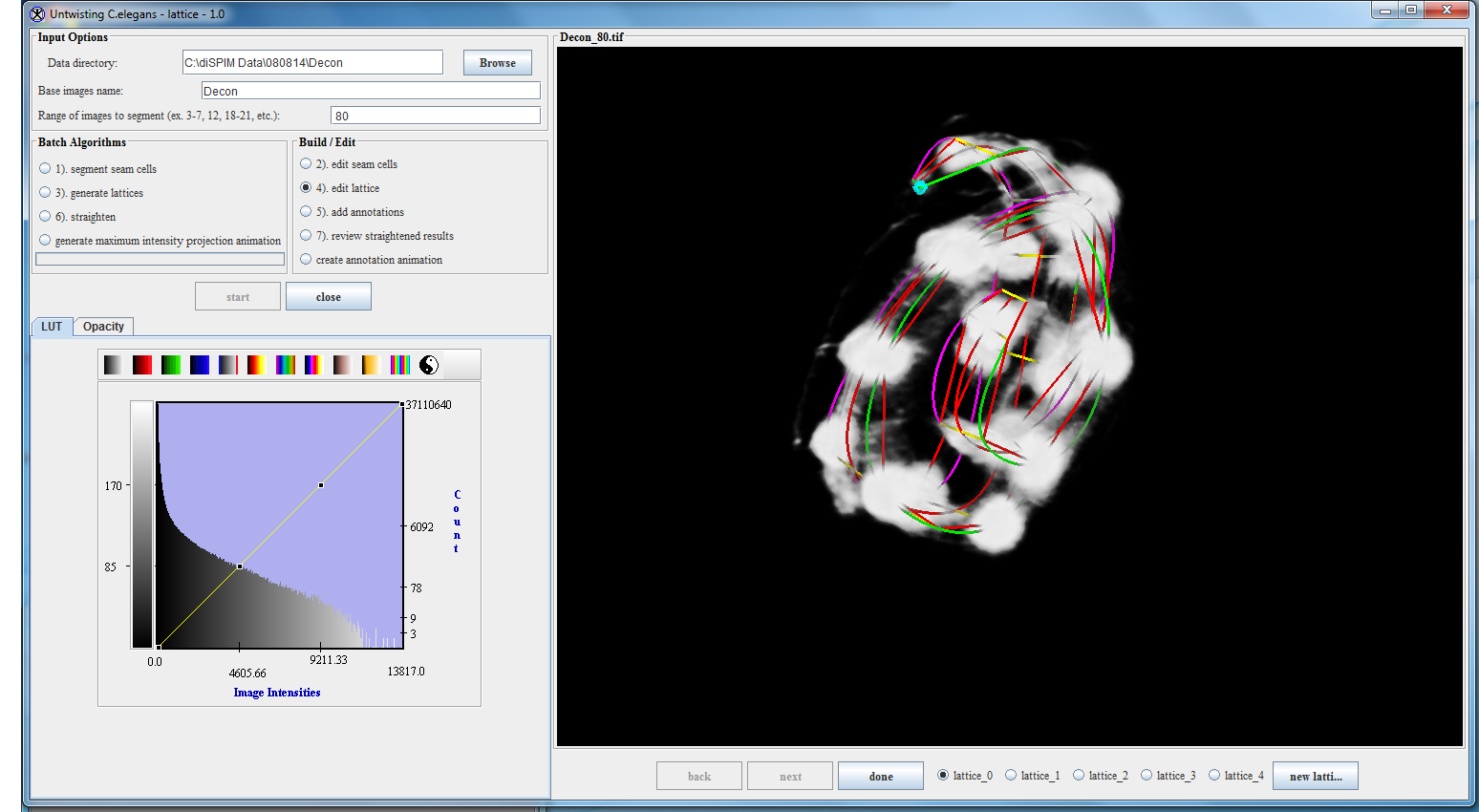


9. In some cases, embryos will either be at too young a stage for automated lattice-building, or none of the automatically-generated lattices will be accurate. In these instances, lattices must be built manually. Begin by selecting the “new lattice” feature. Lattice points can be added by control-clicking on a point in the volume-renderer window. Place the first pair of points on the nose of the embryo:


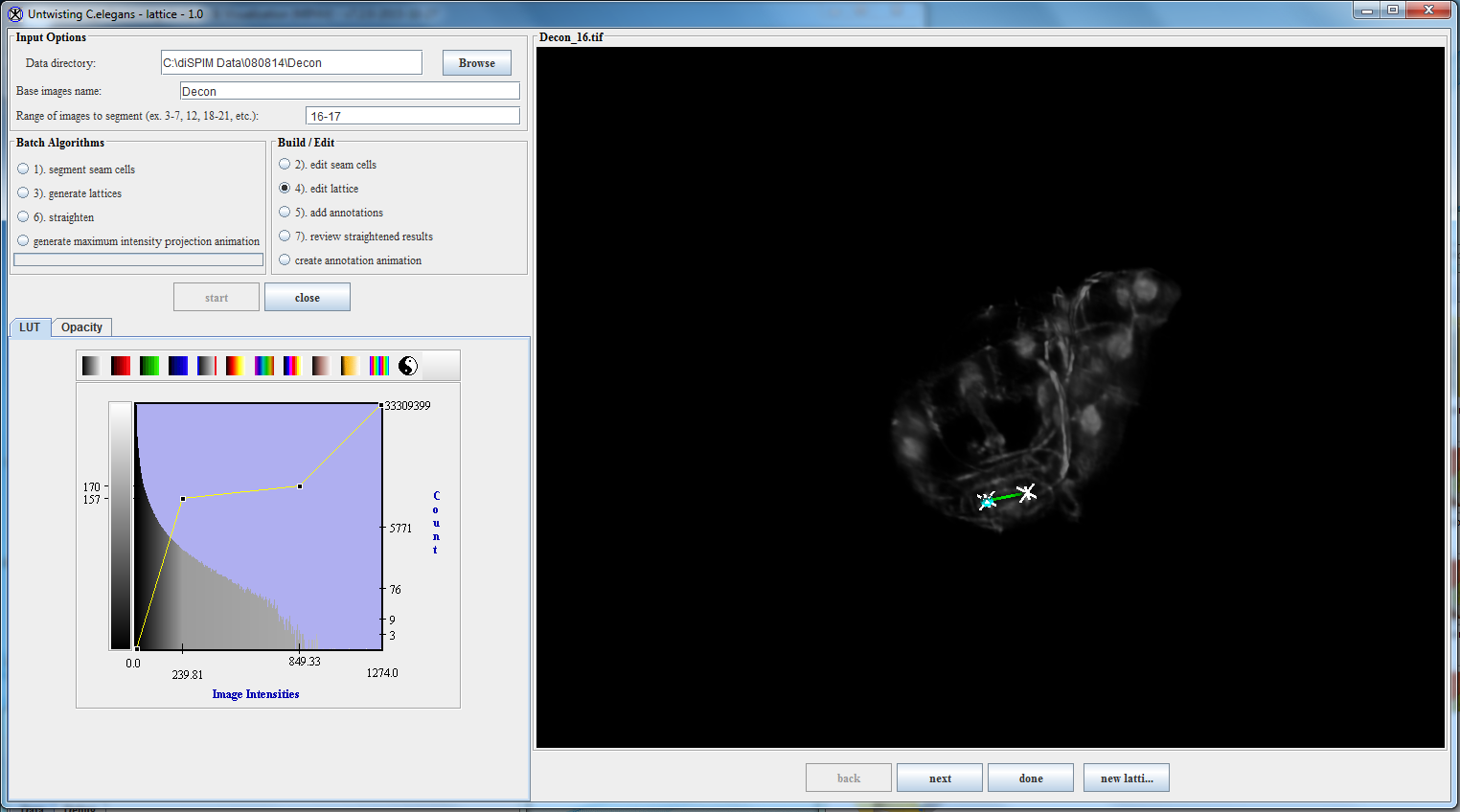


10. Continue adding lattice points in a left-right fashion until you reach the tail of the worm. Once built, the lattice can be edited as for automatically-generated lattices. Subsequent steps are shared between automatically-generated and manually-built lattices. A fully edited hand-built lattice is shown below:


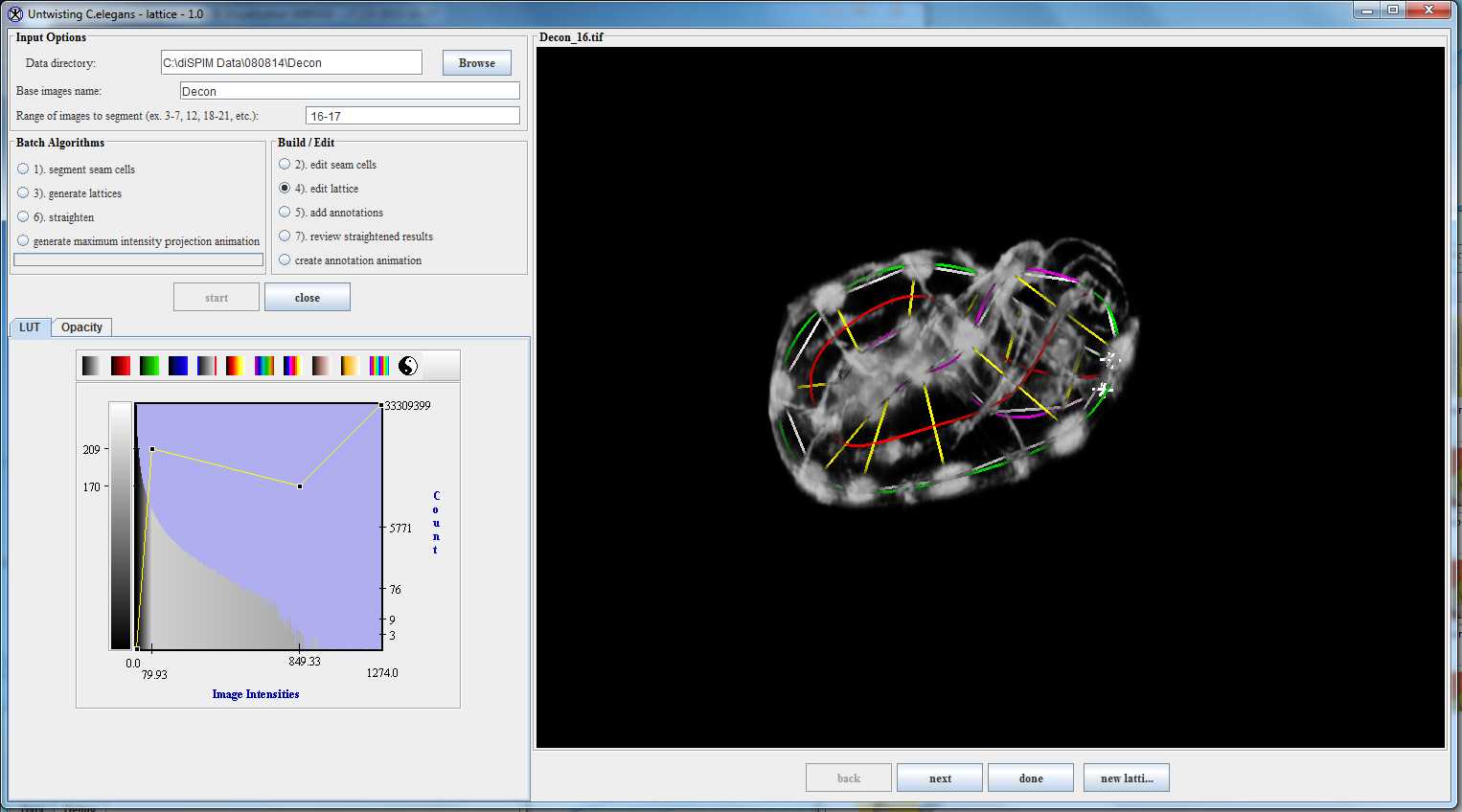


11. If desired, you can then select 5), add annotations, to add annotation points that define locations at which the plugin reports the 3D position after untwisting. The annotation points are added similarly to lattice points, by control-clicking on the location the point should be placed. The 3D position of the annotation points is written to a spreadsheet file after the volumes have been untwisted. When working with an image sequence, the “back” and “next” buttons are used to navigate through the sequence. An annotated embryo is shown below:


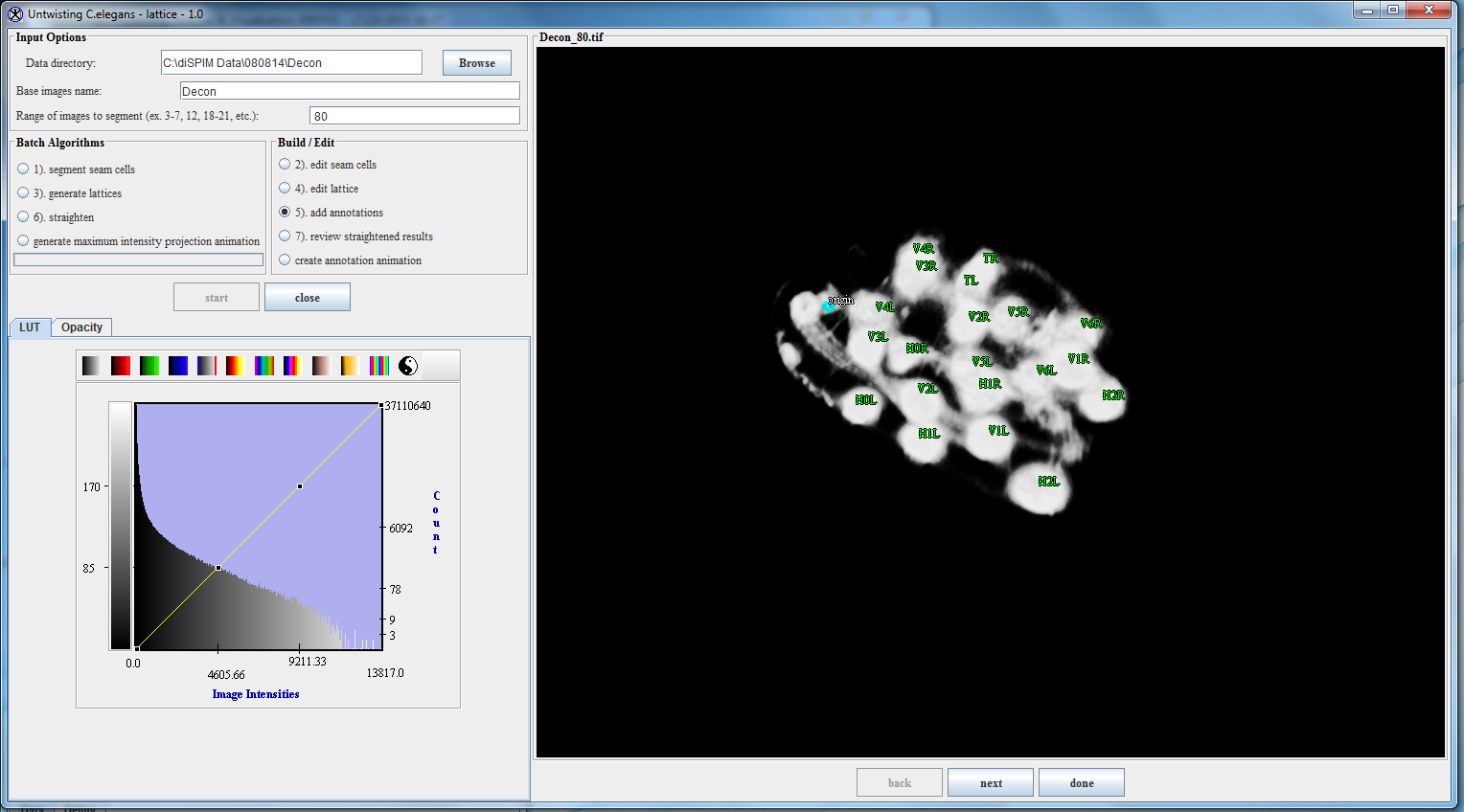


12. After lattices have been generated for each volume, and annotation points added, then you can select 6), straighten, to untwist all the volumes in the sequence.

13. Finally, you can inspect the straightened images using 7), review straightened results. This option displays the straightened volumes, along with associated lattices and annotation points, in the Volume Renderer window. Here the volumes can be inspected, but not edited. As before, the “back” and “next” buttons can be used to move through the images in the sequence. An example of straightened volume inspection is as follows:


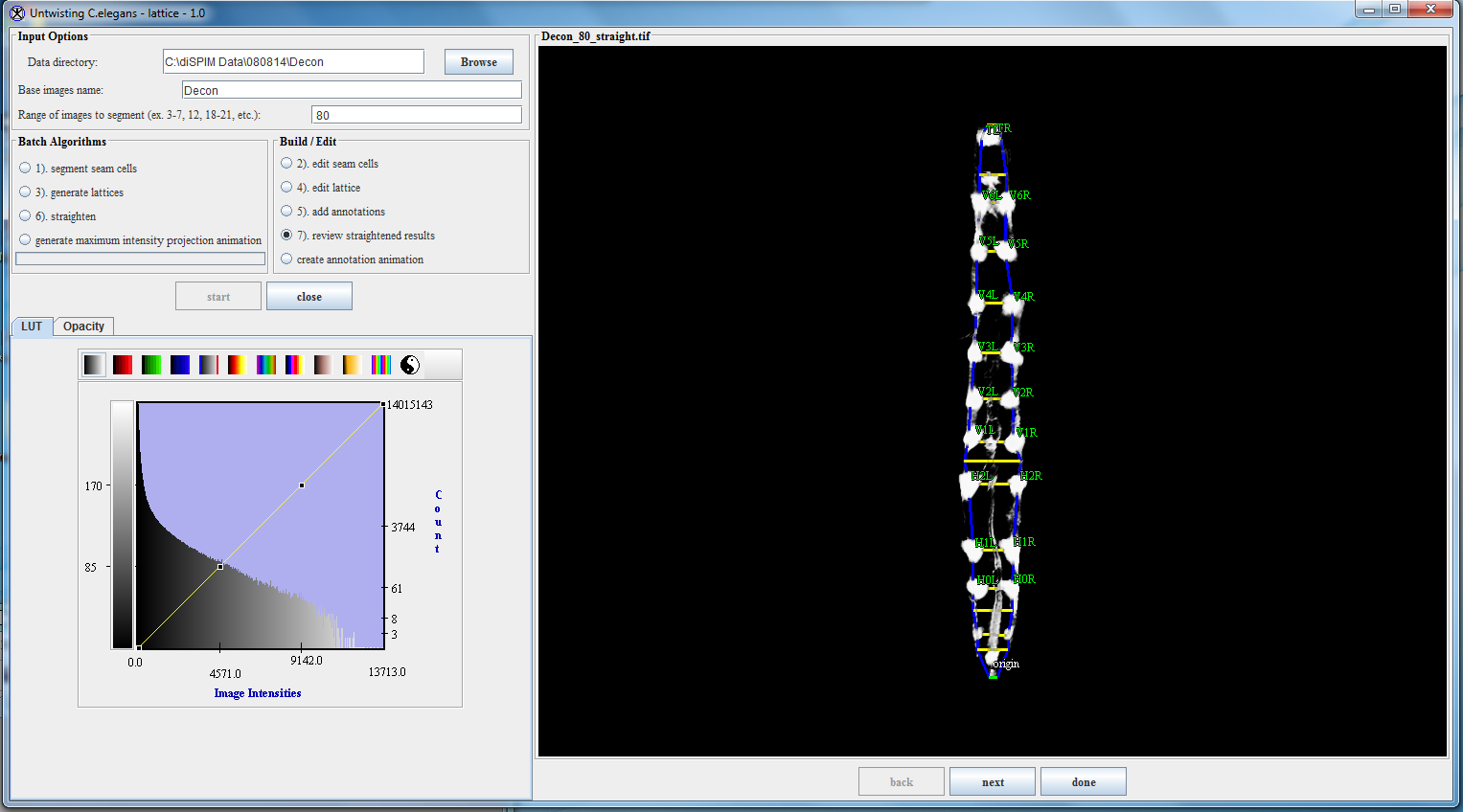


**Additional Options – generate maximum intensity projection animation:**

1. After the straightened volumes have been inspected and you are satisfied that the untwisting is accurate, you can use this option to create a max projection animation (movie) showing all the untwisted volumes. The file path set-up is the same as for untwisting, as the plugin automatically locates the appropriate files. An example of the options used to generate a max intensity animation for three consecutive untwisted volumes is as follows:


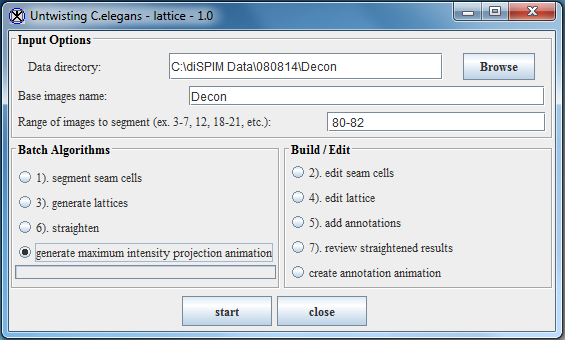


2. Upon pressing ‘start’ ImageJ is opened and the max intensity projections are displayed as part of a stack. The stack can then be saved as an .avi file to create a movie of how the untwisted embryo changes over time. An example of the stack in ImageJ is:


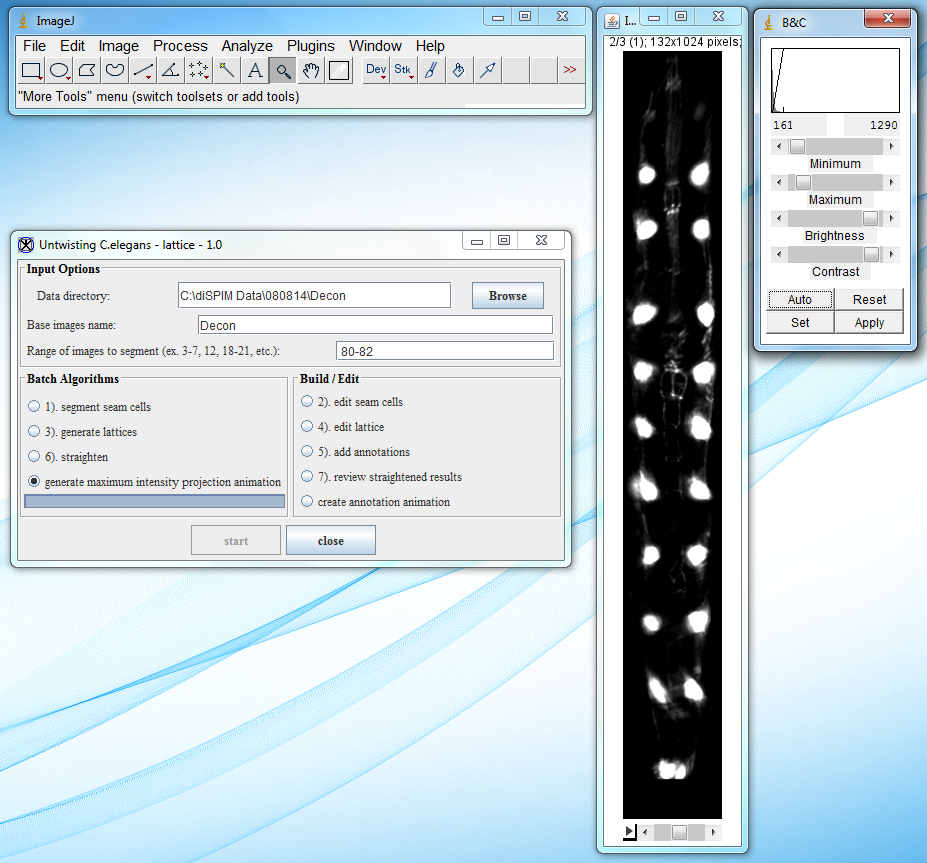


**Additional Options – create annotation animation:**

1. This component creates 3D renderings showing how the position of a cell or cells changes over time, using the position information generated from the annotation capability in the WormUntwisting plugin.

2. WormUntwisting writes the annotation information into a csv (with prefix ‘AnnotationInfo_after’) file in the ‘statistics’ folder. This file lists the 3D position of all annotation points in that volume. Such a spreadsheet appears as:


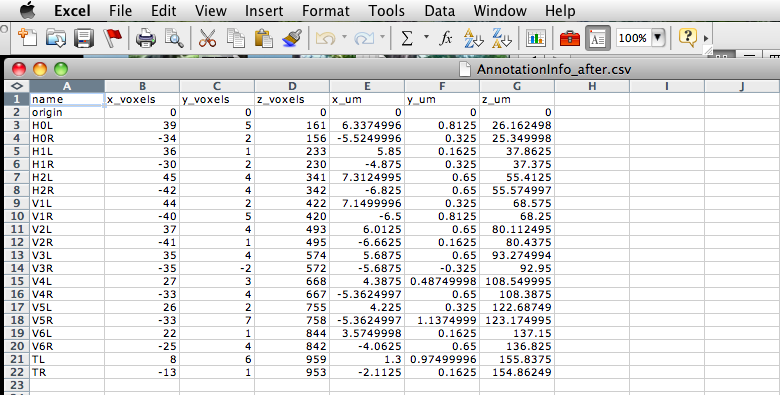


3. Before the annotation_animation component can be run, the 3D position information must be converted from the volume-based ordering (seen in step 2), to a cell-based ordering, where each spreadsheet file consists of 3D positional information for a single annotation point, shown by time. Such a file is shown below, for the H0R seam cell nucleus:


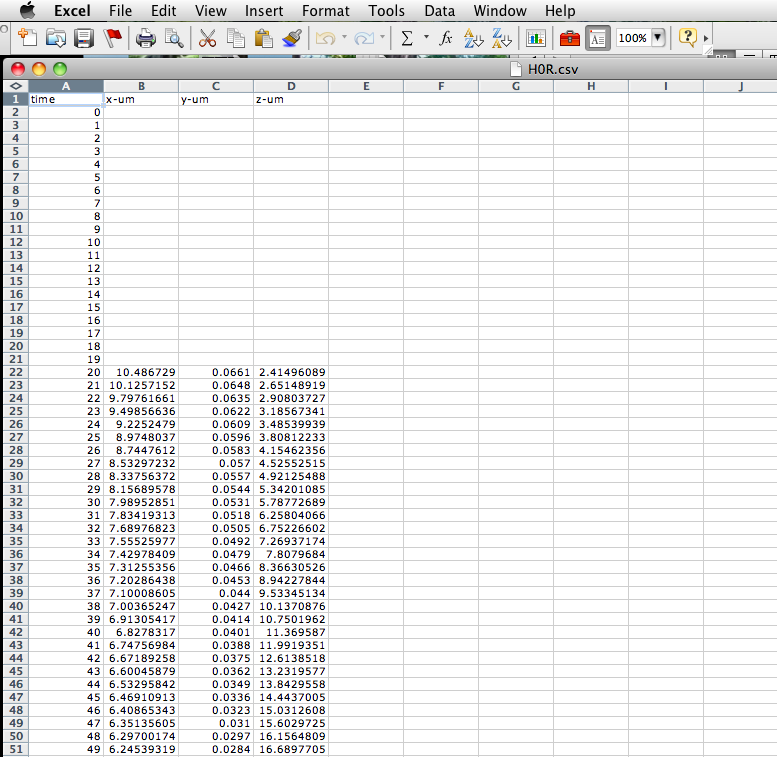


4. After the annotation information is converted to a cell-specific, time-based format you should place all annotation points to be rendered in a single folder, as shown below:


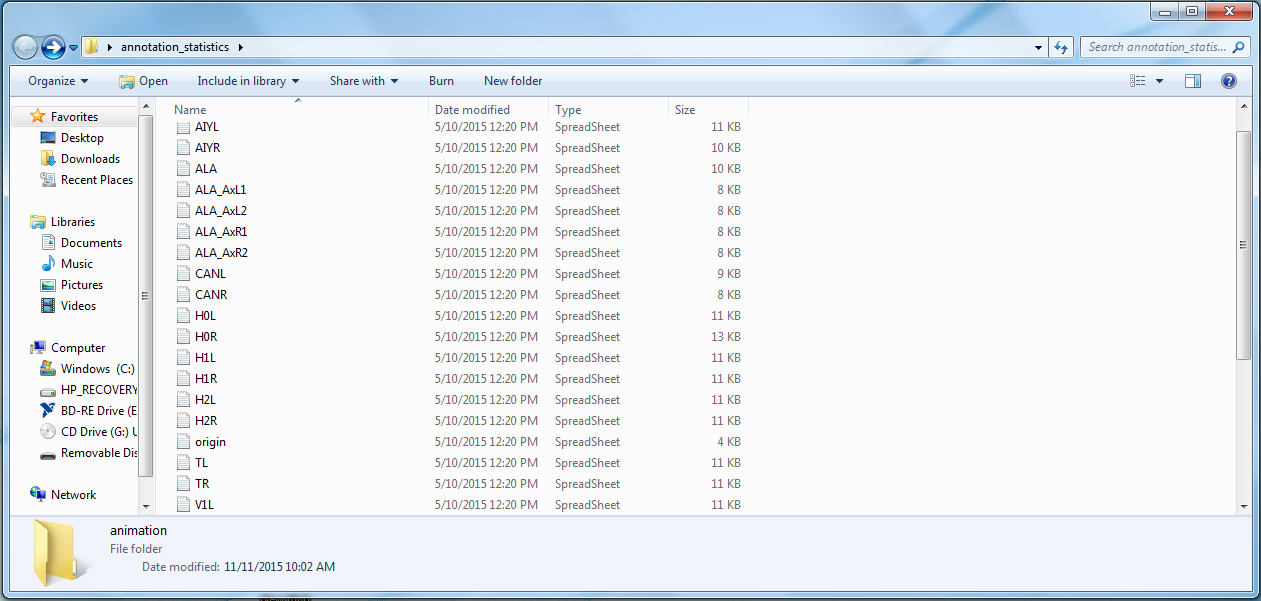


5. You can then open the WormUntwisting plugin, select the folder containing the annotation spreadsheets, and select the annotation_animation option:


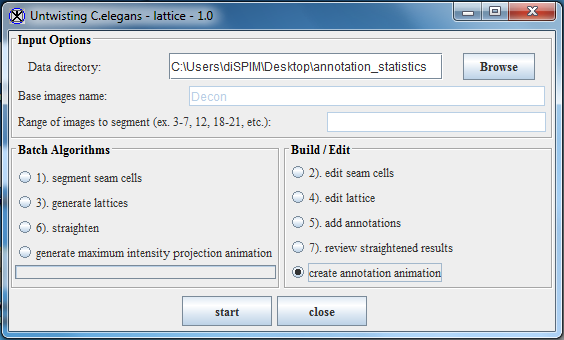


6. The plugin opens a new window showing the rendered annotation points, as well as several control options:


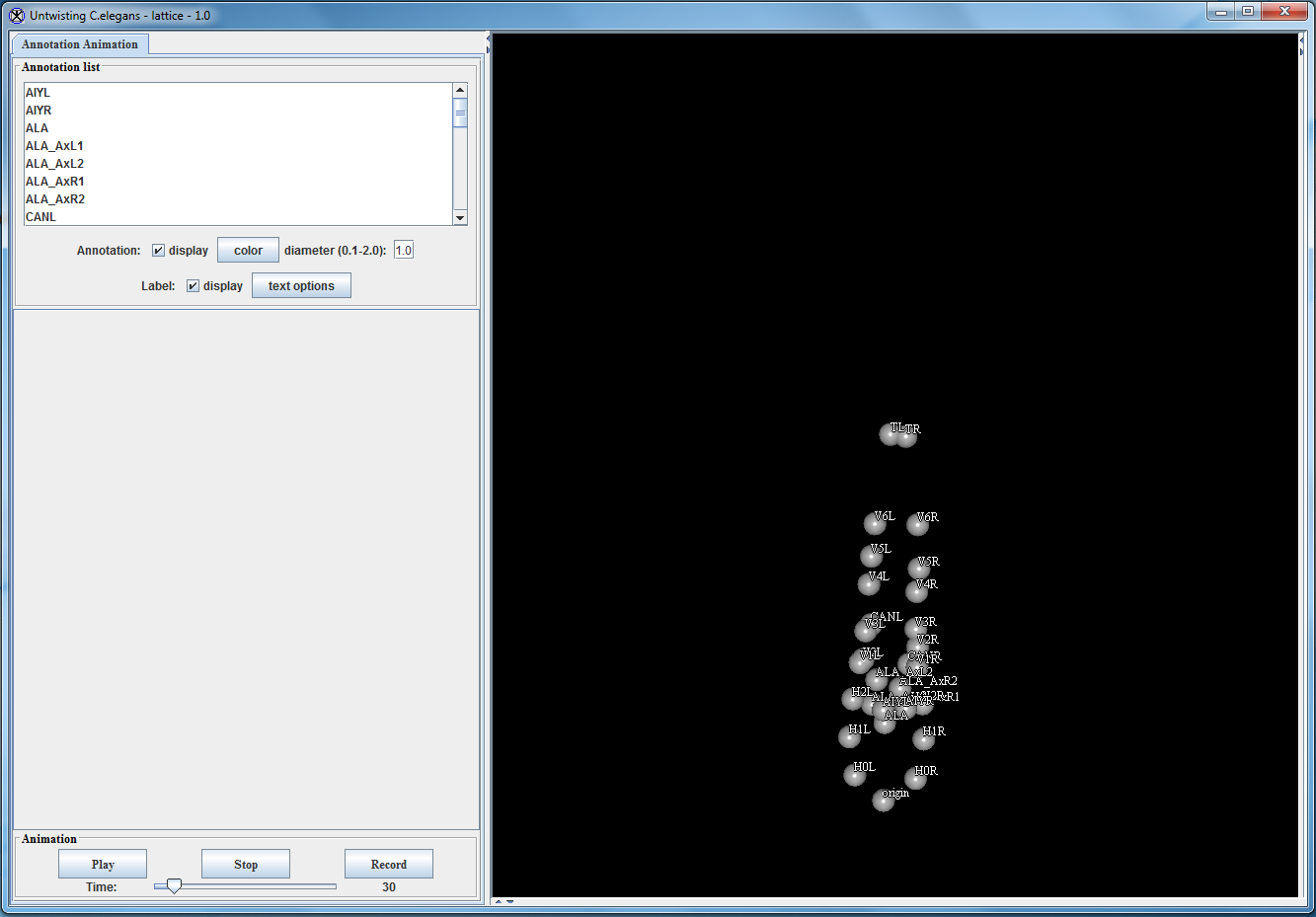


The right-hand side of the window is used for volume rendering similar to that used for untwisting; here the user can rotate and zoom in on the rendering. The bottom left corner has temporal control information, allowing the user to move through a time sequence, and record a movie of the animation as an image sequence. The upper left corner has options to change the appearance of the rendering, including changing text size and color, and the size and color of the rendered points themselves.

7. The user changes the appearance of the rendered points until they are satisfied with the rendering. In the case below, the coloration of rendered points corresponding to neurons was changed to light blue-green:


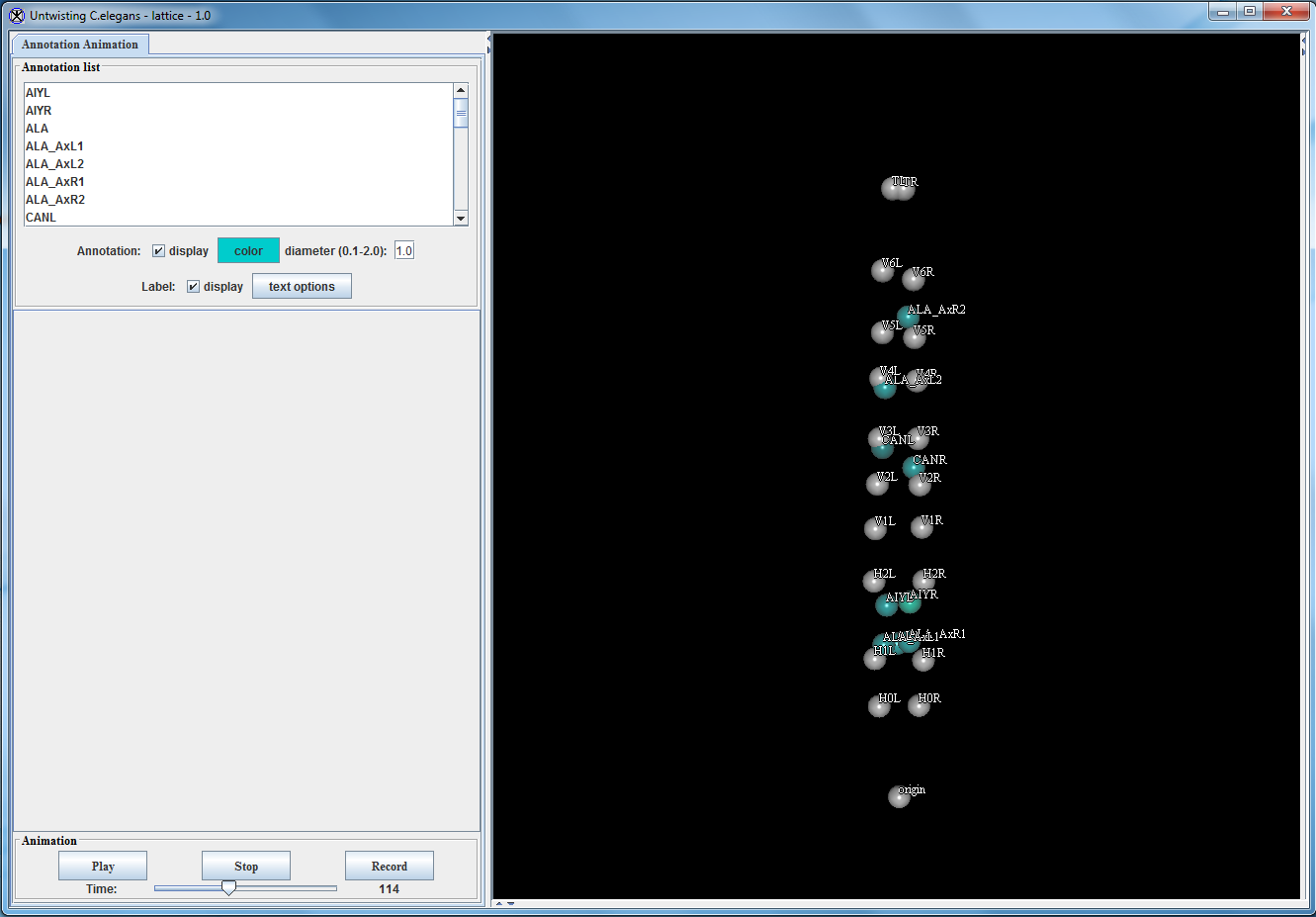


The user can then hit the ‘record’ button in the lower left to record an annotation animation in the form of an image sequence, with each image corresponding to a single time point in the movie. The image sequence can then be opened in a program like ImageJ, saved in .avi format, and used in presentations or publications.
