# Supplementary material for: Untwisting the Caenorhabditis elegans embryo
Source: eLife. 2015 Dec 3;4:e10070. doi: 10.7554/eLife.10070 (PMC4764590; doi:10.7554/eLife.10070)
Supplement: Supplementary file 2. — For each cell studied in this paper, data from 5 embryos were shifted as discussed in the text, and the standard deviations between embryo positions at each timepoint computed. Mean standard deviations (<σ>) and the maximum standard deviation (Max(σ)) over all timepoints are recorded above. For x and y coordinates, the majority of embryo positions are within 2 μm. For z coordinates, most embryo positions lie within 10 μm of each other, with the exception of CANL. See also Figure 4—figure supplement 3 for representative embryo trajectories. For most data displayed here, at least three embryo datasets were used in generating these values. For three datasets (red italics), only two embryo datasets were compared. DOI: http://dx.doi.org/10.7554/eLife.10070.035 [file elife-10070-supp2.docx]

| Cell | <σ_X_>, μm | Max(σ_X_), μm | <σ_Y_>, μm | Max(σ_Y_), μm | <σ_Z_>, μm | Max(σ_Z_), μm |
| --- | --- | --- | --- | --- | --- | --- |
| H0R | 0.9 | 5.2 | 0.7 | 1.4 | 1.8 | 3.8 |
| H0L | 0.7 | 2.0 | 0.6 | 2.5 | 1.8 | 4.0 |
| H1R | 0.8 | 2.2 | 0.7 | 1.9 | 2.3 | 5.9 |
| H1L | 0.8 | 1.4 | 0.7 | 4.2 | 2.5 | 6.2 |
| H2R | 0.8 | 1.8 | 0.7 | 1.6 | 3.1 | 8.4 |
| H2L | 0.9 | 1.9 | 0.7 | 1.8 | 2.7 | 6.4 |
| V1R | 0.9 | 2.0 | 0.7 | 1.5 | 3.4 | 7.7 |
| V1L | 0.8 | 2.1 | 0.7 | 2.0 | 2.9 | 7.0 |
| V2R | 0.8 | 2.2 | 0.7 | 2.0 | 3.6 | 7.5 |
| V2L | 0.8 | 1.9 | 0.6 | 1.7 | 4.8 | 8.4 |
| V3R | 0.8 | 1.6 | 0.7 | 1.3 | 5.3 | 11.5 |
| V3L | 0.8 | 1.7 | 0.7 | 2.0 | 4.6 | 8.5 |
| V4R | 0.8 | 1.8 | 0.6 | 1.3 | 4.7 | 9.0 |
| V4L | 0.8 | 1.8 | 0.6 | 1.7 | 6.9 | 16.6 |
| Q/V5R | 0.8 | 2.0 | 0.7 | 1.5 | 7.2 | 13.1 |
| Q/V5L | 0.8 | 4.1 | 0.8 | 2.9 | 5.5 | 11.1 |
| V6R | 0.6 | 1.4 | 0.7 | 1.6 | 7.5 | 16.3 |
| V6L | 0.7 | 1.2 | 0.7 | 1.5 | 7.8 | 15.1 |
| TR | 0.7 | 6.0 | 0.8 | 5.4 | 6.5 | 27.7 |
| TL | 0.5 | 1.0 | 0.7 | 1.6 | 6.2 | 10.9 |
| *CANR* | *0.8* | *2.4* | *1.3* | *4.8* | *7.5* | *19.1* |
| *CANL* | *0.8* | *3.0* | *1.8* | *4.5* | *22.9* | *33.7* |
| AIYR | 1.0 | 2.3 | 1.0 | 3.2 | 2.8 | 5.6 |
| AIYL | 1.1 | 2.2 | 1.2 | 3.1 | 3.8 | 8.1 |
| *ALA* | *1.2* | *3.4* | *1.1* | *7.0* | *2.3* | *10.0* |
