# Supplementary material for: Untwisting the Caenorhabditis elegans embryo
Source: eLife. 2015 Dec 3;4:e10070. doi: 10.7554/eLife.10070 (PMC4764590; doi:10.7554/eLife.10070)
Supplement: Supplementary file 3. — For each cell studied in this paper, the absolute differences between averaged coordinates and fits were computed at each time point. The means and standard deviations of these differences over time, in μm, are recorded in the table above. For x and y coordinates, the majority of fitted points lie within 1.5 μm of the averaged data, regardless of cell type. For z coordinates, the majority of fitted points lie within 7.5 μm of the averaged data, with the exception of CANL. DOI: http://dx.doi.org/10.7554/eLife.10070.036 [file elife-10070-supp3.docx]

| Cell | μ_\|xavg-xfit\| time_,  μm | σ_\|xavg-xfit\| time_,  μm | μ _\|yavg-yfit\| time_, μm | σ _\|yavg-yfit\| time_, μm | μ _\|zavg-zfit\| time_, μm | σ _\|zavg-zfit\| time_, μm |
| --- | --- | --- | --- | --- | --- | --- |
| H0R | 0.5 | 0.5 | 0.5 | 0.4 | 1.4 | 1.2 |
| H0L | 0.4 | 0.3 | 0.5 | 0.4 | 1.3 | 1.1 |
| H1R | 0.6 | 0.5 | 0.5 | 0.4 | 1.9 | 1.5 |
| H1L | 0.5 | 0.4 | 0.6 | 0.6 | 2.2 | 1.7 |
| H2R | 0.5 | 0.4 | 0.5 | 0.5 | 2.1 | 1.8 |
| H2L | 0.5 | 0.4 | 0.5 | 0.5 | 2.2 | 1.8 |
| V1R | 0.5 | 0.4 | 0.5 | 0.5 | 2.6 | 2.1 |
| V1L | 0.5 | 0.4 | 0.5 | 0.5 | 2.5 | 2.0 |
| V2R | 0.5 | 0.5 | 0.5 | 0.4 | 3.0 | 2.4 |
| V2L | 0.5 | 0.4 | 0.5 | 0.5 | 2.7 | 2.3 |
| V3R | 0.5 | 0.5 | 0.5 | 0.4 | 2.9 | 2.5 |
| V3L | 0.4 | 0.4 | 0.4 | 0.4 | 3.0 | 2.3 |
| V4R | 0.4 | 0.4 | 0.4 | 0.4 | 3.7 | 3.5 |
| V4L | 0.5 | 0.5 | 0.4 | 0.4 | 3.8 | 3.7 |
| Q/V5R | 0.5 | 0.4 | 0.5 | 0.4 | 3.6 | 3.5 |
| Q/V5L | 0.5 | 0.4 | 0.5 | 0.5 | 4.4 | 4.1 |
| V6R | 0.5 | 0.4 | 0.5 | 0.5 | 4.6 | 4.2 |
| V6L | 0.6 | 0.6 | 0.6 | 0.6 | 6.1 | 6.1 |
| TR | 0.4 | 0.4 | 0.4 | 0.4 | 7.3 | 5.7 |
| TL | 0.3 | 0.3 | 0.4 | 0.3 | 7.0 | 5.2 |
| CANR | 0.6 | 0.5 | 0.7 | 0.6 | 6.9 | 4.9 |
| CANL | 0.6 | 0.5 | 0.8 | 0.6 | 10.2 | 9.3 |
| AIYR | 0.7 | 0.6 | 0.9 | 0.8 | 2.6 | 2.0 |
| AIYL | 0.9 | 0.9 | 0.9 | 1.3 | 2.6 | 1.8 |
| ALA | 0.8 | 0.6 | 0.9 | 1.0 | 2.0 | 1.7 |
